# The Significance of Peri-Renal Suppuration

**Published:** 1909-07-17

**Authors:** 


### Surgery.

### THE SIGNIFICANCE OF PERI-RENAL SUPPURATION.

A peri-renal abscess is not in itself a clinical entity. It should be regarded rather in the light of a symptom manifesting itself in the course of disease attacking the kidney itself or some adjacent, or even remote, viscus; in other words it is almost always a secondary phenomenon, and a primary focus should be sought for.

It is, of course, often connected with disease of the kidney itself; and there may be found a fistulous track leading from a septic focus in the kidney to the areolar connective tissue which surrounds the organ; but, in other cases, no such macroscopic communication can be found, the infection having spread through an apparently intact renal capsule. The commonest renal condition which may cause a peri-renal abscess is a renal calculus, round which a small focus of suppuration has made its appearance.

When the disease starts in the kidney the diagnosis is not likely to be difficult, because attention is already focussed on that organ by the original symptoms of the calculus: thus blood and pus will have been found in the urine, and the presence of a calculus may already have been demonstrated by means of *x*-rays. What is difficult to know is the exact moment at which the suppuration has extended from the kidney into the peri-renal tissues. But then, again, this is not a point of great clinical importance, since an operation is demanded as soon as the existence of a calculous pyonephrosis has been made certain, and to postpone an operation at that time would be bad surgical practice.

But there are also a number of conditions, starting in organs remote from the kidney itself, which may give rise to a peri-renal abscess; and in these the exact diagnosis may give rise to greater difficulty because no indication of disease may be given until an abscess has formed round the kidney. The most prominent of these are conditions connected with the alimentary tract. First and foremost comes suppuration in connection with the appendix. When an abscess forms in or round an appendix which lies retrocæcally it may make its way upwards behind the ascending colon, lying entirely behind the peritoneum, and the abscess may eventually surround the kidney, and if left point in the loin. Or again a gastric ulcer may slowly leak and form a peri-gastric abscess which will spread into the peri-renal tissues, and an abscess in connection with malignant disease of the ascending or descending colon may do the same thing. Of the remoter causes an empyema which makes its way through the diaphragm, and a parametric abscess which spreads up behind the posterior layer of the broad ligament are the commonest.

The symptoms which distinguish a peri-renal abscess, apart from those due to the primary disease, are a constant dull pain in the affected loin with pyrexia, and those general manifestations that one associates with a collection of pus locked up in the body, namely malaise, anorexia and headache. On examining the abdomen the muscles of the affected side are found to be held somewhat rigid, and there is tenderness on palpation. A mass may or may not be felt, but its outlines are never definite, because the pus is contained in a loose areolar space, in which it can spread with comparative ease. There is often œdema of the skin of the corresponding loin, but hardly ever, except in the latest stages of an untreated case, a red fluctuating swelling. For it must be remembered that the pus is deep to the deep fascia, and as long as that is so, those cardinal signs of acute inflammation, redness of the skin and the presence of a fluctuating swelling will be wanting. This is equally true, whatever part of the body is affected. Thus a patient may have a deep popliteal abscess, but it is only when the pus has made its way through the deep fascia that a red, fluctuating swelling will be found.

There are, however, two symptoms which, though not constantly observed, are, when present, highly suggestive. One is flexion of and the other is œdema in the corresponding limb. The flexion is, of course, easily explained. It is due to an irritative contraction of the psoas muscle, which lies directly behind the kidney. The œdema is due either to pressure on the common iliac vein by the abscess, or to thrombosis set up by the adjacent suppuration.

In the early stages the treatment is usually confined to symptomatic remedies such as local counter-irritation by the application of hot fomentations to the loin and antipyretic drugs; and the common teaching is that no operation should be performed until it is certain that an abscess is present. If, however, there is any doubt as to this, the casting vote should, in the writer's opinion, always be given in favour of operation. Œdema of the loin should be regarded as a positive indication for surgical interference. For then it may be confidently predicted that even if pus is not actually found at the operation, it will make its appearance at an early date. At the operation an attempt should always be made to investigate the origin of the pus; so that it may be known what measures, if any, must be taken subsequently. At the first operation all that is necessary is to provide efficient drainage. For this purpose one or more large india-rubber tubes should be inserted into the wound, which should be partially sewn up. If adequate drainage is provided the condition should gradually clear up whether the original cause was appendicitis or empyema; and when the discharge has diminished or ceased the removal of the appendix or resection of a rib can be considered later.

On the other hand, the consequences of postponing surgical interference when an abscess has once formed may be grave. For the pus will surely spread along the lines of least resistance, either backwards into the loin, downwards into the pelvis, or upwards into the lesser sac of peritoneum, or even through the diaphragm, and so cause an empyema where none was before.

